# Supplementary material for: HIVprotI: an integrated web based platform for prediction and design of HIV proteins inhibitors
Source: J Cheminform. 2018 Mar 9;10:12. doi: 10.1186/s13321-018-0266-y (PMC5845081; doi:10.1186/s13321-018-0266-y)
Supplement: Supplementary file 2 — Additional file 2. Source code of HIVProtI web server. [file 13321_2018_266_MOESM2_ESM.zip › HIVprotI_Source-code/hivprotI/weka-3-6-6/doc/weka/core/Instances.html]

Instances


---


|  |  |  |
| --- | --- | --- |
| **Overview** \| **Package** \| **Class** \| **Tree** \| **Deprecated** \| **Index** \| **Help** \| **Weka's home** | |  |
| **PREV CLASS**   **NEXT CLASS** | **FRAMES**    **NO FRAMES**     **All Classes** |  |
| SUMMARY: NESTED \| FIELD \| CONSTR \| METHOD | DETAIL: FIELD \| CONSTR \| METHOD |  |


---


## weka.core Class Instances

```
java.lang.Object
  weka.core.Instances
```

**All Implemented Interfaces:**: java.io.Serializable, RevisionHandler

**Direct Known Subclasses:**: IndividualInstances, ReferenceInstances

---

``` public class Instances extends java.lang.Object implements java.io.Serializable, RevisionHandler ```

Class for handling an ordered set of weighted instances.

Typical usage:

```
 import weka.core.converters.ConverterUtils.DataSource;
 ...

 // Read all the instances in the file (ARFF, CSV, XRFF, ...)
 DataSource source = new DataSource(filename);
 Instances instances = source.getDataSet();

 // Make the last attribute be the class
 instances.setClassIndex(instances.numAttributes() - 1);

 // Print header and instances.
 System.out.println("\nDataset:\n");
 System.out.println(instances);

 ...
```

All methods that change a set of instances are safe, ie. a change
of a set of instances does not affect any other sets of
instances. All methods that change a datasets's attribute
information clone the dataset before it is changed.

**Version:**
:   $Revision: 6996 $

**Author:**
:   Eibe Frank (eibe@cs.waikato.ac.nz), Len Trigg (trigg@cs.waikato.ac.nz), FracPete (fracpete at waikato dot ac dot nz)

**See Also:**: Serialized Form

---

| **Field Summary** | |
| --- | --- |
| `static java.lang.String` | `ARFF_DATA`             The keyword used to denote the start of the arff data section |
| `static java.lang.String` | `ARFF_RELATION`             The keyword used to denote the start of an arff header |
| `static java.lang.String` | `FILE_EXTENSION`             The filename extension that should be used for arff files |
| `static java.lang.String` | `SERIALIZED_OBJ_FILE_EXTENSION`             The filename extension that should be used for bin. |


**Constructor Summary**

`Instances(Instances dataset)`
  
          Constructor copying all instances and references to
the header information from the given set of instances.

`Instances(Instances dataset,
int capacity)`
  
          Constructor creating an empty set of instances.

`Instances(Instances source,
int first,
int toCopy)`
  
          Creates a new set of instances by copying a
subset of another set.

`Instances(java.io.Reader reader)`
  
          Reads an ARFF file from a reader, and assigns a weight of
one to each instance.

`Instances(java.io.Reader reader,
int capacity)`
  
          **Deprecated.** *instead of using this method in conjunction with the
`readInstance(Reader)` method, one should use the
`ArffLoader` or `DataSource` class instead.*

`Instances(java.lang.String name,
FastVector attInfo,
int capacity)`
  
          Creates an empty set of instances.


| **Method Summary** | |
| --- | --- |
| `void` | `add(Instance instance)`             Adds one instance to the end of the set. |
| `Attribute` | `attribute(int index)`             Returns an attribute. |
| `Attribute` | `attribute(java.lang.String name)`             Returns an attribute given its name. |
| `AttributeStats` | `attributeStats(int index)`             Calculates summary statistics on the values that appear in this set of instances for a specified attribute. |
| `double[]` | `attributeToDoubleArray(int index)`             Gets the value of all instances in this dataset for a particular attribute. |
| `boolean` | `checkForAttributeType(int attType)`             Checks for attributes of the given type in the dataset |
| `boolean` | `checkForStringAttributes()`             Checks for string attributes in the dataset |
| `boolean` | `checkInstance(Instance instance)`             Checks if the given instance is compatible with this dataset. |
| `Attribute` | `classAttribute()`             Returns the class attribute. |
| `int` | `classIndex()`             Returns the class attribute's index. |
| `void` | `compactify()`             Compactifies the set of instances. |
| `void` | `delete()`             Removes all instances from the set. |
| `void` | `delete(int index)`             Removes an instance at the given position from the set. |
| `void` | `deleteAttributeAt(int position)`             Deletes an attribute at the given position (0 to numAttributes() - 1). |
| `void` | `deleteAttributeType(int attType)`             Deletes all attributes of the given type in the dataset. |
| `void` | `deleteStringAttributes()`             Deletes all string attributes in the dataset. |
| `void` | `deleteWithMissing(Attribute att)`             Removes all instances with missing values for a particular attribute from the dataset. |
| `void` | `deleteWithMissing(int attIndex)`             Removes all instances with missing values for a particular attribute from the dataset. |
| `void` | `deleteWithMissingClass()`             Removes all instances with a missing class value from the dataset. |
| `java.util.Enumeration` | `enumerateAttributes()`             Returns an enumeration of all the attributes. |
| `java.util.Enumeration` | `enumerateInstances()`             Returns an enumeration of all instances in the dataset. |
| `boolean` | `equalHeaders(Instances dataset)`             Checks if two headers are equivalent. |
| `Instance` | `firstInstance()`             Returns the first instance in the set. |
| `java.util.Random` | `getRandomNumberGenerator(long seed)`             Returns a random number generator. |
| `java.lang.String` | `getRevision()`             Returns the revision string. |
| `void` | `insertAttributeAt(Attribute att, int position)`             Inserts an attribute at the given position (0 to numAttributes()) and sets all values to be missing. |
| `Instance` | `instance(int index)`             Returns the instance at the given position. |
| `double` | `kthSmallestValue(Attribute att, int k)`             Returns the kth-smallest attribute value of a numeric attribute. |
| `double` | `kthSmallestValue(int attIndex, int k)`             Returns the kth-smallest attribute value of a numeric attribute. |
| `Instance` | `lastInstance()`             Returns the last instance in the set. |
| `static void` | `main(java.lang.String[] args)`             Main method for this class. |
| `double` | `meanOrMode(Attribute att)`             Returns the mean (mode) for a numeric (nominal) attribute as a floating-point value. |
| `double` | `meanOrMode(int attIndex)`             Returns the mean (mode) for a numeric (nominal) attribute as a floating-point value. |
| `static Instances` | `mergeInstances(Instances first, Instances second)`             Merges two sets of Instances together. |
| `int` | `numAttributes()`             Returns the number of attributes. |
| `int` | `numClasses()`             Returns the number of class labels. |
| `int` | `numDistinctValues(Attribute att)`             Returns the number of distinct values of a given attribute. |
| `int` | `numDistinctValues(int attIndex)`             Returns the number of distinct values of a given attribute. |
| `int` | `numInstances()`             Returns the number of instances in the dataset. |
| `void` | `randomize(java.util.Random random)`             Shuffles the instances in the set so that they are ordered randomly. |
| `boolean` | `readInstance(java.io.Reader reader)`             **Deprecated.** *instead of using this method in conjunction with the `readInstance(Reader)` method, one should use the `ArffLoader` or `DataSource` class instead.* |
| `java.lang.String` | `relationName()`             Returns the relation's name. |
| `void` | `renameAttribute(Attribute att, java.lang.String name)`             Renames an attribute. |
| `void` | `renameAttribute(int att, java.lang.String name)`             Renames an attribute. |
| `void` | `renameAttributeValue(Attribute att, java.lang.String val, java.lang.String name)`             Renames the value of a nominal (or string) attribute value. |
| `void` | `renameAttributeValue(int att, int val, java.lang.String name)`             Renames the value of a nominal (or string) attribute value. |
| `Instances` | `resample(java.util.Random random)`             Creates a new dataset of the same size using random sampling with replacement. |
| `Instances` | `resampleWithWeights(java.util.Random random)`             Creates a new dataset of the same size using random sampling with replacement according to the current instance weights. |
| `Instances` | `resampleWithWeights(java.util.Random random, double[] weights)`             Creates a new dataset of the same size using random sampling with replacement according to the given weight vector. |
| `void` | `setClass(Attribute att)`             Sets the class attribute. |
| `void` | `setClassIndex(int classIndex)`             Sets the class index of the set. |
| `void` | `setRelationName(java.lang.String newName)`             Sets the relation's name. |
| `void` | `sort(Attribute att)`             Sorts the instances based on an attribute. |
| `void` | `sort(int attIndex)`             Sorts the instances based on an attribute. |
| `void` | `stratify(int numFolds)`             Stratifies a set of instances according to its class values if the class attribute is nominal (so that afterwards a stratified cross-validation can be performed). |
| `Instances` | `stringFreeStructure()`             Create a copy of the structure if the data has string or relational attributes, "cleanses" string types (i.e. |
| `double` | `sumOfWeights()`             Computes the sum of all the instances' weights. |
| `void` | `swap(int i, int j)`             Swaps two instances in the set. |
| `static void` | `test(java.lang.String[] argv)`             Method for testing this class. |
| `Instances` | `testCV(int numFolds, int numFold)`             Creates the test set for one fold of a cross-validation on the dataset. |
| `java.lang.String` | `toString()`             Returns the dataset as a string in ARFF format. |
| `java.lang.String` | `toSummaryString()`             Generates a string summarizing the set of instances. |
| `Instances` | `trainCV(int numFolds, int numFold)`             Creates the training set for one fold of a cross-validation on the dataset. |
| `Instances` | `trainCV(int numFolds, int numFold, java.util.Random random)`             Creates the training set for one fold of a cross-validation on the dataset. |
| `double` | `variance(Attribute att)`             Computes the variance for a numeric attribute. |
| `double` | `variance(int attIndex)`             Computes the variance for a numeric attribute. |

**Methods inherited from class java.lang.Object**

`equals, getClass, hashCode, notify, notifyAll, wait, wait, wait`

**Field Detail**

### FILE\_EXTENSION

```
public static final java.lang.String FILE_EXTENSION
```

:   The filename extension that should be used for arff files

    **See Also:**: Constant Field Values

---


### SERIALIZED\_OBJ\_FILE\_EXTENSION

```
public static final java.lang.String SERIALIZED_OBJ_FILE_EXTENSION
```

:   The filename extension that should be used for bin. serialized instances files

    **See Also:**: Constant Field Values

---


### ARFF\_RELATION

```
public static final java.lang.String ARFF_RELATION
```

:   The keyword used to denote the start of an arff header

    **See Also:**: Constant Field Values

---


### ARFF\_DATA

```
public static final java.lang.String ARFF_DATA
```

:   The keyword used to denote the start of the arff data section

    **See Also:**: Constant Field Values


**Constructor Detail**

### Instances

```
public Instances(java.io.Reader reader)
          throws java.io.IOException
```

:   Reads an ARFF file from a reader, and assigns a weight of
    one to each instance. Lets the index of the class
    attribute be undefined (negative).

    **Parameters:**: `reader` - the reader **Throws:**: `java.io.IOException` - if the ARFF file is not read successfully

---


### Instances

```
@Deprecated
public Instances(java.io.Reader reader,
                            int capacity)
          throws java.io.IOException
```

:   **Deprecated.** *instead of using this method in conjunction with the
    `readInstance(Reader)` method, one should use the
    `ArffLoader` or `DataSource` class instead.*

    :   Reads the header of an ARFF file from a reader and
        reserves space for the given number of instances. Lets
        the class index be undefined (negative).

        **Parameters:**: `reader` - the reader: `capacity` - the capacity **Throws:**: `java.lang.IllegalArgumentException` - if the header is not read successfully or the capacity is negative.: `java.io.IOException` - if there is a problem with the reader. **See Also:**: `ArffLoader`, `ConverterUtils.DataSource`

---


### Instances

```
public Instances(Instances dataset)
```

:   Constructor copying all instances and references to
    the header information from the given set of instances.

    **Parameters:**: `dataset` - the set to be copied

---


### Instances

```
public Instances(Instances dataset,
                 int capacity)
```

:   Constructor creating an empty set of instances. Copies references
    to the header information from the given set of instances. Sets
    the capacity of the set of instances to 0 if its negative.

    **Parameters:**: `dataset` - the instances from which the header information is to be taken: `capacity` - the capacity of the new dataset

---


### Instances

```
public Instances(Instances source,
                 int first,
                 int toCopy)
```

:   Creates a new set of instances by copying a
    subset of another set.

    **Parameters:**: `source` - the set of instances from which a subset is to be created: `first` - the index of the first instance to be copied: `toCopy` - the number of instances to be copied **Throws:**: `java.lang.IllegalArgumentException` - if first and toCopy are out of range

---


### Instances

```
public Instances(java.lang.String name,
                 FastVector attInfo,
                 int capacity)
```

:   Creates an empty set of instances. Uses the given
    attribute information. Sets the capacity of the set of
    instances to 0 if its negative. Given attribute information
    must not be changed after this constructor has been used.

    **Parameters:**: `name` - the name of the relation: `attInfo` - the attribute information: `capacity` - the capacity of the set


**Method Detail**

### stringFreeStructure

```
public Instances stringFreeStructure()
```

:   Create a copy of the structure if the data has string or
    relational attributes, "cleanses" string types (i.e. doesn't
    contain references to the strings seen in the past) and all
    relational attributes.

    :   **Returns:**: a copy of the instance structure.

---


### add

```
public void add(Instance instance)
```

:   Adds one instance to the end of the set.
    Shallow copies instance before it is added. Increases the
    size of the dataset if it is not large enough. Does not
    check if the instance is compatible with the dataset.
    Note: String or relational values are not transferred.

    :   **Parameters:**: `instance` - the instance to be added

---


### attribute

```
public Attribute attribute(int index)
```

:   Returns an attribute.

    :   **Parameters:**: `index` - the attribute's index (index starts with 0) **Returns:**: the attribute at the given position

---


### attribute

```
public Attribute attribute(java.lang.String name)
```

:   Returns an attribute given its name. If there is more than
    one attribute with the same name, it returns the first one.
    Returns null if the attribute can't be found.

    :   **Parameters:**: `name` - the attribute's name **Returns:**: the attribute with the given name, null if the attribute can't be found

---


### checkForAttributeType

```
public boolean checkForAttributeType(int attType)
```

:   Checks for attributes of the given type in the dataset

    :   **Parameters:**: `attType` - the attribute type to look for **Returns:**: true if attributes of the given type are present

---


### checkForStringAttributes

```
public boolean checkForStringAttributes()
```

:   Checks for string attributes in the dataset

    :   **Returns:**: true if string attributes are present, false otherwise

---


### checkInstance

```
public boolean checkInstance(Instance instance)
```

:   Checks if the given instance is compatible
    with this dataset. Only looks at the size of
    the instance and the ranges of the values for
    nominal and string attributes.

    :   **Parameters:**: `instance` - the instance to check **Returns:**: true if the instance is compatible with the dataset

---


### classAttribute

```
public Attribute classAttribute()
```

:   Returns the class attribute.

    :   **Returns:**: the class attribute **Throws:**: `UnassignedClassException` - if the class is not set

---


### classIndex

```
public int classIndex()
```

:   Returns the class attribute's index. Returns negative number
    if it's undefined.

    :   **Returns:**: the class index as an integer

---


### compactify

```
public void compactify()
```

:   Compactifies the set of instances. Decreases the capacity of
    the set so that it matches the number of instances in the set.

---


### delete

```
public void delete()
```

:   Removes all instances from the set.

---


### delete

```
public void delete(int index)
```

:   Removes an instance at the given position from the set.

    :   **Parameters:**: `index` - the instance's position (index starts with 0)

---


### deleteAttributeAt

```
public void deleteAttributeAt(int position)
```

:   Deletes an attribute at the given position
    (0 to numAttributes() - 1). A deep copy of the attribute
    information is performed before the attribute is deleted.

    :   **Parameters:**: `position` - the attribute's position (position starts with 0) **Throws:**: `java.lang.IllegalArgumentException` - if the given index is out of range or the class attribute is being deleted

---


### deleteAttributeType

```
public void deleteAttributeType(int attType)
```

:   Deletes all attributes of the given type in the dataset. A deep copy of
    the attribute information is performed before an attribute is deleted.

    :   **Parameters:**: `attType` - the attribute type to delete **Throws:**: `java.lang.IllegalArgumentException` - if attribute couldn't be successfully deleted (probably because it is the class attribute).

---


### deleteStringAttributes

```
public void deleteStringAttributes()
```

:   Deletes all string attributes in the dataset. A deep copy of the attribute
    information is performed before an attribute is deleted.

    :   **Throws:**: `java.lang.IllegalArgumentException` - if string attribute couldn't be successfully deleted (probably because it is the class attribute). **See Also:**: `deleteAttributeType(int)`

---


### deleteWithMissing

```
public void deleteWithMissing(int attIndex)
```

:   Removes all instances with missing values for a particular
    attribute from the dataset.

    :   **Parameters:**: `attIndex` - the attribute's index (index starts with 0)

---


### deleteWithMissing

```
public void deleteWithMissing(Attribute att)
```

:   Removes all instances with missing values for a particular
    attribute from the dataset.

    :   **Parameters:**: `att` - the attribute

---


### deleteWithMissingClass

```
public void deleteWithMissingClass()
```

:   Removes all instances with a missing class value
    from the dataset.

    :   **Throws:**: `UnassignedClassException` - if class is not set

---


### enumerateAttributes

```
public java.util.Enumeration enumerateAttributes()
```

:   Returns an enumeration of all the attributes.

    :   **Returns:**: enumeration of all the attributes.

---


### enumerateInstances

```
public java.util.Enumeration enumerateInstances()
```

:   Returns an enumeration of all instances in the dataset.

    :   **Returns:**: enumeration of all instances in the dataset

---


### equalHeaders

```
public boolean equalHeaders(Instances dataset)
```

:   Checks if two headers are equivalent.

    :   **Parameters:**: `dataset` - another dataset **Returns:**: true if the header of the given dataset is equivalent to this header

---


### firstInstance

```
public Instance firstInstance()
```

:   Returns the first instance in the set.

    :   **Returns:**: the first instance in the set

---


### getRandomNumberGenerator

```
public java.util.Random getRandomNumberGenerator(long seed)
```

:   Returns a random number generator. The initial seed of the random
    number generator depends on the given seed and the hash code of
    a string representation of a instances chosen based on the given
    seed.

    :   **Parameters:**: `seed` - the given seed **Returns:**: the random number generator

---


### insertAttributeAt

```
public void insertAttributeAt(Attribute att,
                              int position)
```

:   Inserts an attribute at the given position (0 to
    numAttributes()) and sets all values to be missing.
    Shallow copies the attribute before it is inserted, and performs
    a deep copy of the existing attribute information.

    :   **Parameters:**: `att` - the attribute to be inserted: `position` - the attribute's position (position starts with 0) **Throws:**: `java.lang.IllegalArgumentException` - if the given index is out of range

---


### instance

```
public Instance instance(int index)
```

:   Returns the instance at the given position.

    :   **Parameters:**: `index` - the instance's index (index starts with 0) **Returns:**: the instance at the given position

---


### kthSmallestValue

```
public double kthSmallestValue(Attribute att,
                               int k)
```

:   Returns the kth-smallest attribute value of a numeric attribute.
    Note that calling this method will change the order of the data!

    :   **Parameters:**: `att` - the Attribute object: `k` - the value of k **Returns:**: the kth-smallest value

---


### kthSmallestValue

```
public double kthSmallestValue(int attIndex,
                               int k)
```

:   Returns the kth-smallest attribute value of a numeric attribute.
    Note that calling this method will change the order of the data!
    The number of non-missing values in the data must be as least
    as last as k for this to work.

    :   **Parameters:**: `attIndex` - the attribute's index: `k` - the value of k **Returns:**: the kth-smallest value

---


### lastInstance

```
public Instance lastInstance()
```

:   Returns the last instance in the set.

    :   **Returns:**: the last instance in the set

---


### meanOrMode

```
public double meanOrMode(int attIndex)
```

:   Returns the mean (mode) for a numeric (nominal) attribute as
    a floating-point value. Returns 0 if the attribute is neither nominal nor
    numeric. If all values are missing it returns zero.

    :   **Parameters:**: `attIndex` - the attribute's index (index starts with 0) **Returns:**: the mean or the mode

---


### meanOrMode

```
public double meanOrMode(Attribute att)
```

:   Returns the mean (mode) for a numeric (nominal) attribute as a
    floating-point value. Returns 0 if the attribute is neither
    nominal nor numeric. If all values are missing it returns zero.

    :   **Parameters:**: `att` - the attribute **Returns:**: the mean or the mode

---


### numAttributes

```
public int numAttributes()
```

:   Returns the number of attributes.

    :   **Returns:**: the number of attributes as an integer

---


### numClasses

```
public int numClasses()
```

:   Returns the number of class labels.

    :   **Returns:**: the number of class labels as an integer if the class attribute is nominal, 1 otherwise. **Throws:**: `UnassignedClassException` - if the class is not set

---


### numDistinctValues

```
public int numDistinctValues(int attIndex)
```

:   Returns the number of distinct values of a given attribute.
    Returns the number of instances if the attribute is a
    string attribute. The value 'missing' is not counted.

    :   **Parameters:**: `attIndex` - the attribute (index starts with 0) **Returns:**: the number of distinct values of a given attribute

---


### numDistinctValues

```
public int numDistinctValues(Attribute att)
```

:   Returns the number of distinct values of a given attribute.
    Returns the number of instances if the attribute is a
    string attribute. The value 'missing' is not counted.

    :   **Parameters:**: `att` - the attribute **Returns:**: the number of distinct values of a given attribute

---


### numInstances

```
public int numInstances()
```

:   Returns the number of instances in the dataset.

    :   **Returns:**: the number of instances in the dataset as an integer

---


### randomize

```
public void randomize(java.util.Random random)
```

:   Shuffles the instances in the set so that they are ordered
    randomly.

    :   **Parameters:**: `random` - a random number generator

---


### readInstance

```
@Deprecated
public boolean readInstance(java.io.Reader reader)
                     throws java.io.IOException
```

:   **Deprecated.** *instead of using this method in conjunction with the
    `readInstance(Reader)` method, one should use the
    `ArffLoader` or `DataSource` class instead.*

    :   Reads a single instance from the reader and appends it
        to the dataset. Automatically expands the dataset if it
        is not large enough to hold the instance. This method does
        not check for carriage return at the end of the line.

        :   **Parameters:**: `reader` - the reader **Returns:**: false if end of file has been reached **Throws:**: `java.io.IOException` - if the information is not read successfully **See Also:**: `ArffLoader`, `ConverterUtils.DataSource`

---


### relationName

```
public java.lang.String relationName()
```

:   Returns the relation's name.

    :   **Returns:**: the relation's name as a string

---


### renameAttribute

```
public void renameAttribute(int att,
                            java.lang.String name)
```

:   Renames an attribute. This change only affects this
    dataset.

    :   **Parameters:**: `att` - the attribute's index (index starts with 0): `name` - the new name

---


### renameAttribute

```
public void renameAttribute(Attribute att,
                            java.lang.String name)
```

:   Renames an attribute. This change only affects this
    dataset.

    :   **Parameters:**: `att` - the attribute: `name` - the new name

---


### renameAttributeValue

```
public void renameAttributeValue(int att,
                                 int val,
                                 java.lang.String name)
```

:   Renames the value of a nominal (or string) attribute value. This
    change only affects this dataset.

    :   **Parameters:**: `att` - the attribute's index (index starts with 0): `val` - the value's index (index starts with 0): `name` - the new name

---


### renameAttributeValue

```
public void renameAttributeValue(Attribute att,
                                 java.lang.String val,
                                 java.lang.String name)
```

:   Renames the value of a nominal (or string) attribute value. This
    change only affects this dataset.

    :   **Parameters:**: `att` - the attribute: `val` - the value: `name` - the new name

---


### resample

```
public Instances resample(java.util.Random random)
```

:   Creates a new dataset of the same size using random sampling
    with replacement.

    :   **Parameters:**: `random` - a random number generator **Returns:**: the new dataset

---


### resampleWithWeights

```
public Instances resampleWithWeights(java.util.Random random)
```

:   Creates a new dataset of the same size using random sampling
    with replacement according to the current instance weights. The
    weights of the instances in the new dataset are set to one.

    :   **Parameters:**: `random` - a random number generator **Returns:**: the new dataset

---


### resampleWithWeights

```
public Instances resampleWithWeights(java.util.Random random,
                                     double[] weights)
```

:   Creates a new dataset of the same size using random sampling
    with replacement according to the given weight vector. The
    weights of the instances in the new dataset are set to one.
    The length of the weight vector has to be the same as the
    number of instances in the dataset, and all weights have to
    be positive.

    :   **Parameters:**: `random` - a random number generator: `weights` - the weight vector **Returns:**: the new dataset **Throws:**: `java.lang.IllegalArgumentException` - if the weights array is of the wrong length or contains negative weights.

---


### setClass

```
public void setClass(Attribute att)
```

:   Sets the class attribute.

    :   **Parameters:**: `att` - attribute to be the class

---


### setClassIndex

```
public void setClassIndex(int classIndex)
```

:   Sets the class index of the set.
    If the class index is negative there is assumed to be no class.
    (ie. it is undefined)

    :   **Parameters:**: `classIndex` - the new class index (index starts with 0) **Throws:**: `java.lang.IllegalArgumentException` - if the class index is too big or < 0

---


### setRelationName

```
public void setRelationName(java.lang.String newName)
```

:   Sets the relation's name.

    :   **Parameters:**: `newName` - the new relation name.

---


### sort

```
public void sort(int attIndex)
```

:   Sorts the instances based on an attribute. For numeric attributes,
    instances are sorted in ascending order. For nominal attributes,
    instances are sorted based on the attribute label ordering
    specified in the header. Instances with missing values for the
    attribute are placed at the end of the dataset.

    :   **Parameters:**: `attIndex` - the attribute's index (index starts with 0)

---


### sort

```
public void sort(Attribute att)
```

:   Sorts the instances based on an attribute. For numeric attributes,
    instances are sorted into ascending order. For nominal attributes,
    instances are sorted based on the attribute label ordering
    specified in the header. Instances with missing values for the
    attribute are placed at the end of the dataset.

    :   **Parameters:**: `att` - the attribute

---


### stratify

```
public void stratify(int numFolds)
```

:   Stratifies a set of instances according to its class values
    if the class attribute is nominal (so that afterwards a
    stratified cross-validation can be performed).

    :   **Parameters:**: `numFolds` - the number of folds in the cross-validation **Throws:**: `UnassignedClassException` - if the class is not set

---


### sumOfWeights

```
public double sumOfWeights()
```

:   Computes the sum of all the instances' weights.

    :   **Returns:**: the sum of all the instances' weights as a double

---


### testCV

```
public Instances testCV(int numFolds,
                        int numFold)
```

:   Creates the test set for one fold of a cross-validation on
    the dataset.

    :   **Parameters:**: `numFolds` - the number of folds in the cross-validation. Must be greater than 1.: `numFold` - 0 for the first fold, 1 for the second, ... **Returns:**: the test set as a set of weighted instances **Throws:**: `java.lang.IllegalArgumentException` - if the number of folds is less than 2 or greater than the number of instances.

---


### toString

```
public java.lang.String toString()
```

:   Returns the dataset as a string in ARFF format. Strings
    are quoted if they contain whitespace characters, or if they
    are a question mark.

    :   **Overrides:**: `toString` in class `java.lang.Object`
    :   **Returns:**: the dataset in ARFF format as a string

---


### trainCV

```
public Instances trainCV(int numFolds,
                         int numFold)
```

:   Creates the training set for one fold of a cross-validation
    on the dataset.

    :   **Parameters:**: `numFolds` - the number of folds in the cross-validation. Must be greater than 1.: `numFold` - 0 for the first fold, 1 for the second, ... **Returns:**: the training set **Throws:**: `java.lang.IllegalArgumentException` - if the number of folds is less than 2 or greater than the number of instances.

---


### trainCV

```
public Instances trainCV(int numFolds,
                         int numFold,
                         java.util.Random random)
```

:   Creates the training set for one fold of a cross-validation
    on the dataset. The data is subsequently randomized based
    on the given random number generator.

    :   **Parameters:**: `numFolds` - the number of folds in the cross-validation. Must be greater than 1.: `numFold` - 0 for the first fold, 1 for the second, ...: `random` - the random number generator **Returns:**: the training set **Throws:**: `java.lang.IllegalArgumentException` - if the number of folds is less than 2 or greater than the number of instances.

---


### variance

```
public double variance(int attIndex)
```

:   Computes the variance for a numeric attribute.

    :   **Parameters:**: `attIndex` - the numeric attribute (index starts with 0) **Returns:**: the variance if the attribute is numeric **Throws:**: `java.lang.IllegalArgumentException` - if the attribute is not numeric

---


### variance

```
public double variance(Attribute att)
```

:   Computes the variance for a numeric attribute.

    :   **Parameters:**: `att` - the numeric attribute **Returns:**: the variance if the attribute is numeric **Throws:**: `java.lang.IllegalArgumentException` - if the attribute is not numeric

---


### attributeStats

```
public AttributeStats attributeStats(int index)
```

:   Calculates summary statistics on the values that appear in this
    set of instances for a specified attribute.

    :   **Parameters:**: `index` - the index of the attribute to summarize (index starts with 0) **Returns:**: an AttributeStats object with it's fields calculated.

---


### attributeToDoubleArray

```
public double[] attributeToDoubleArray(int index)
```

:   Gets the value of all instances in this dataset for a particular
    attribute. Useful in conjunction with Utils.sort to allow iterating
    through the dataset in sorted order for some attribute.

    :   **Parameters:**: `index` - the index of the attribute. **Returns:**: an array containing the value of the desired attribute for each instance in the dataset.

---


### toSummaryString

```
public java.lang.String toSummaryString()
```

:   Generates a string summarizing the set of instances. Gives a breakdown
    for each attribute indicating the number of missing/discrete/unique
    values and other information.

    :   **Returns:**: a string summarizing the dataset

---


### swap

```
public void swap(int i,
                 int j)
```

:   Swaps two instances in the set.

    :   **Parameters:**: `i` - the first instance's index (index starts with 0): `j` - the second instance's index (index starts with 0)

---


### mergeInstances

```
public static Instances mergeInstances(Instances first,
                                       Instances second)
```

:   Merges two sets of Instances together. The resulting set will have
    all the attributes of the first set plus all the attributes of the
    second set. The number of instances in both sets must be the same.

    :   **Parameters:**: `first` - the first set of Instances: `second` - the second set of Instances **Returns:**: the merged set of Instances **Throws:**: `java.lang.IllegalArgumentException` - if the datasets are not the same size

---


### test

```
public static void test(java.lang.String[] argv)
```

:   Method for testing this class.

    :   **Parameters:**: `argv` - should contain one element: the name of an ARFF file

---


### main

```
public static void main(java.lang.String[] args)
```

:   Main method for this class. The following calls are possible:

    - `weka.core.Instances` help
    - `weka.core.Instances` <filename>
    - `weka.core.Instances` merge <filename1> <filename2>
    - `weka.core.Instances` append <filename1> <filename2>
    - `weka.core.Instances` headers <filename1> <filename2>
    - `weka.core.Instances` randomize <seed> <filename>

    :   **Parameters:**: `args` - the commandline parameters

---


### getRevision

```
public java.lang.String getRevision()
```

:   Returns the revision string.

    :   **Specified by:**: `getRevision` in interface `RevisionHandler`
    :   **Returns:**: the revision


---


|  |  |  |
| --- | --- | --- |
| **Overview** \| **Package** \| **Class** \| **Tree** \| **Deprecated** \| **Index** \| **Help** \| **Weka's home** | |  |
| **PREV CLASS**   **NEXT CLASS** | **FRAMES**    **NO FRAMES**     **All Classes** |  |
| SUMMARY: NESTED \| FIELD \| CONSTR \| METHOD | DETAIL: FIELD \| CONSTR \| METHOD |  |


---
